# Supplementary material for: MAT-PointPillars: Enhanced PointPillars algorithm based on multi-scale attention mechanisms and transformer
Source: PLoS One. 2025 Jun 27;20(6):e0325373. doi: 10.1371/journal.pone.0325373 (PMC12204542; doi:10.1371/journal.pone.0325373)
Supplement: Attachment — Submitted filename: Response_to_Decision_Letter_auresp_2.docx [file pone.0325373.s003.docx]

# Dear Reviewers,

Thank you for your valuable comments on our submitted manuscripts. We are aware that your feedback is essential for enhancing the quality and standard of our work. We have carefully considered and reflected on the various suggestions made by you and have revised the article. The specific responses are as follows:

# Reviewer #3 ：

1. **Can you try to explain in the article why we need to use multi-scale attention mechanisms and what are the benefits of multi-scale attention.**

**Reply:** Thank you for your suggestion. Multi-scale attention mechanisms are essential for capturing features at different scales, which is particularly important for detecting objects of varying sizes in 3D point clouds. Different scales use different attention mechanisms, and different scales can obtain different features. Small targets like cyclists often have sparse and irregular point distributions, making them challenging to detect. By incorporating multi-scale attention, the network can focus on both local details and global context, improving detection accuracy.

2. **Can you explain why CMBA Attention, CA Attention, and Transform are used in three different scale branches? Have you tried using one attention mechanism or changing the order for all of them.**

**Reply:** Thank you for your suggestion. We refer to TPH-YOLOv5, the small-scale feature extraction module based on Transformer integrates the Transformer Encoder and the upsampling module to enhance the network’s ability to extract small-scale features. CBAM (Convolutional Block Attention Module) and CA (Coordinate Attention) are used in the first and second stages to enhance spatial and channel-wise feature extraction for larger-scale objects. As shown in Table 5, in the ablation experiment, we conduct separate ablation experiments on key submodules such as CBAM and CA. For example, we remove the CBAM and CA modules, respectively, and observe the changes in model performance.

**Table 5.** Results of ablation experiment

| Attention | Batch | Head | Layer | AP3D | APBEV |
| --- | --- | --- | --- | --- | --- |
| CBAM+CBAM | 2 | 2 | 1 | 78.40% | 81.07% |
| CBAM+CBAM | 2 | 4 | 1 | 76.41% | 79.93% |
| CA+CA | 2 | 4 | 2 | 73.47% | 78.91% |
| CA+CBAM | 2 | 4 | 2 | 74.98% | 78.00% |
| CBAM+CBAM | 2 | 4 | 2 | 81.15% | 83.85% |

3. **Please maintain consistency in the font of the text, caption, and main text in the image, such as Figure 4 and Figure 8.**

**Reply:** Thank you for your suggestion. We acknowledge the inconsistency in fonts and will ensure uniformity across all figures and captions in the revised manuscript. The figures and captions will be standardized to match the main text font.

4. **Add citations to the following papers: "Towards Generalizable Person Re-identification with a Bi-stream Generative Model" and "Mix-Modality Person Re-Identification: A New and Practical Paradigm".**

**Reply:** Thank you for your suggestion. We will include citations to the suggested papers in the "Introduction" section to broaden the discussion on multi-modal and generative approaches. The relevant content has been added in the text. Please refer to the highlighted part.

# Reviewer #4:

1. **Explain how the solution guarantees desired system performance?**

**Reply:** Thank you for your suggestion. The proposed MAT-PointPillars algorithm ensures desired system performance through a combination of multi-scale attention mechanisms and Transformer Encoder integration, which enhance feature extraction and detection accuracy while maintaining real-time processing capabilities. By introducing the Attention Deconv module in the first and second stages of the backbone network, the algorithm improves the extraction of large-scale semantic information, while the Transformer Deconv module in the third stage enhances small-scale feature extraction, particularly for challenging targets like cyclists. As Fig 10 indicates, the system operates at an average frame rate of 22.63 fps, exceeding the sampling frequency of conventional LiDAR(10Hz), thus meeting real-time requirements.

FPS

|  | MVA-PP | Pointpillar |
| --- | --- | --- |
| 0 | 19 | 20 |
| 1 | 25 | 25 |
| 2 | 21 | 20 |
| 3 | 24 | 24 |
| 4 | 24 | 22 |
| 5 | 20 | 20 |
| 6 | 24 | 25 |
| 7 | 21 | 19 |
| 8 | 25 | 24 |
| 9 | 24 | 22 |
| 10 | 20 | 23 |
| 11 | 24 | 25 |
| 12 | 20 | 20 |
| 13 | 24 | 25 |
| 14 | 21 | 19 |
| 15 | 22 | 22 |
| 16 | 24 | 25 |
| 17 | 20 | 19 |
| 18 | 24 | 24 |
| 19 | 22 | 20 |
| 20 | 23 | 23 |
| 21 | 25 | 25 |
| 22 | 21 | 20 |
| 23 | 25 | 25 |
| 24 | 20 | 18 |

**Fig 10.** Line chart of frame rate

2. **What is the advantage in this article in relation to existing methodologies? Discussions and explanations should be provided on this issue.**

**Reply:** Thank you for your suggestion. The proposed algorithm demonstrates significant advantages over existing methodologies in several key aspects. Firstly, it achieves superior detection accuracy, particularly for small and irregular targets like cyclists, with AP3D improvements of 2.44%, 1.19%, and 1.23% across easy, moderate, and hard difficulty levels compared to the baseline PointPillars model. Additionally, the algorithm maintains real-time performance with an average frame rate of 22.63 fps, surpassing conventional LiDAR sampling frequencies, thus balancing accuracy and efficiency. The ablation experiments further validate the effectiveness of the proposed modules. Overall, MAT-PointPillars addresses the limitations of existing methods by combining multi-scale feature extraction, attention mechanisms, and Transformer-based enhancements, resulting in robust detection capabilities for small targets and real-world applications.

3. **Comparative experiments are suggested to demonstrate the superiority of the proposed approach. And more discussions should be given to clearly demonstrate the effectiveness of the obtained results.**

**Reply:** Thank you for your suggestion. To demonstrate the superiority of MAT-PointPillars, comparative experiments were conducted against leading 3D object detection methods, including VoxelNet, PillarNet, SECOND, and the baseline PointPillars. The results show that MAT-PointPillars achieves the highest AP3D scores (81.15%, 62.02%, and 58.68% for easy, moderate, and hard cases, respectively), outperforming PointPillars by 2.44%, 1.19%, and 1.23%, and surpassing other methods in both 3D and BEV detection accuracy. The improvements are particularly notable for small targets like cyclists. These experiments and discussions clearly validate that MAT-PointPillars offers a balanced and superior solution for 3D object detection, especially in challenging scenarios with small or occluded objects.

4. **The difficulty encountered in this research should also be discussed in order to show the present research is not a trivial extension of existing methodologies.**

**Reply:** Thank you for your suggestion. The research faces two major challenges. First, detecting small and sparse targets (e.g., cyclists) in point clouds is inherently difficult due to limited geometric features and occlusions. To address this, we integrate vision-inspired Transformer Encoders into the 3D detection framework, enabling the model to capture long-range dependencies and enrich semantic context for sparse point clusters—an approach not previously explored in PointPillars-based methods. Second, maintaining real-time performance while incorporating computationally intensive modules posed a significant hurdle. We optimize the system by carefully balancing model depth and efficiency, deploying it on ROS with a streamlined pipeline that achieved 22.63 fps—faster than conventional LiDAR sampling rates. These solutions highlight the non-trivial contributions of the work, bridging the gap between accuracy and real-time feasibility in 3D object detection.

5. **The literature review of topic considered in the paper should be better structured. It should lead to introducing what the motivation of this paper is, what has not been done before and why the authors addressed and studied this system structure.**

**Reply:** Thank you for your suggestion. Existing 3D object detection methods, such as voxel-based (VoxelNet, SECOND) and pillar-based (PointPillars) approaches, struggle with accurately detecting small and irregular targets like cyclists due to sparse point cloud data and limited feature representation. While attention mechanisms and multi-scale feature fusion have been explored in 2D vision (e.g., CBAM, Transformer), their direct application to 3D point clouds remains underexplored, especially in balancing real-time performance. Previous works either lacked global context modeling (e.g., PointPillars) or introduced excessive computational overhead (e.g., PV-RCNN), failing to achieve both high accuracy and efficiency. This paper addresses these gaps by proposing MAT-PointPillars, which integrates multi-scale attention (CBAM/CA) and Transformer Encoders to enhance small-target detection.

6. **It is unclear how original the study is in terms of theoretical advancements, further information on this matter is needed. The Introduction section needs to include a few references to emphasize the contribution: Enhanced Feature Extraction YOLO Industrial Small Object Detection Algorithm based on Receptive-Field Attention and Multi-scale Features, Measurement Science and Technology; End-to-end multi-scale residual network with parallel attention mechanism for fault diagnosis under noise and small samples, ISA Transactions; Quantized Iterative Learning Control of Communication Constrained System with the Encoding and Decoding Mechanism, Transactions of the Institute of Measurement and Control; and in that way point out additional modern strategies and opportunities. Please be aware that your text will be more up-to-date if your references are up-to-date.**

**Reply:** Thank you for your suggestion. We will include citations to the suggested papers in the "Introduction" section to broaden the discussion on multi-modal and generative approaches. The relevant content has been added in the text. Please refer to the highlighted part.

7. **A consistent style for included references should be followed, and every information should be verified. It is crucial to verify all cited papers, fill in any gaps with Volumes, Issues, and Pages, and fix any inaccurate data. In addition, DOI numbers must be provided to any current references that have not yet been appeared in the Volume and Issue.**

**Reply:** Thank you for your suggestion. The manuscript will ensure that all references adhere to a consistent citation style, every detail will be verified for accuracy. And provide the DOI number for the current reference.
