# Supplementary material for: MAT-PointPillars: Enhanced PointPillars algorithm based on multi-scale attention mechanisms and transformer
Source: PLoS One. 2025 Jun 27;20(6):e0325373. doi: 10.1371/journal.pone.0325373 (PMC12204542; doi:10.1371/journal.pone.0325373)
Supplement: Attachment — Submitted filename: Response to Decision Letter.docx [file pone.0325373.s002.docx]

# Dear Reviewers,

Thank you for your valuable comments on our submitted manuscripts. We are aware that your feedback is essential for enhancing the quality and standard of our work. We have carefully considered and reflected on the various suggestions made by you and have revised the article. The specific responses are as follows:

# Reviewer #1 and Reviewer #2:

1. **Insufficient number of comparison methods in the article. There are only two comparison methods on the KITTI dataset. / Insufficient number of comparison methods.**

**Reply:** Thank you very much for your suggestion. A more comprehensive comparison is essential to demonstrate the effectiveness of our proposed method. In response to your comment, we have expanded the experimental evaluation by adding two additional methods on the KITTI dataset. Specifically, we included comparisons with PillarNet and SECOND, as shown in Table 4 of the revised manuscript. These new comparisons provide a more robust and thorough analysis of our method's performance relative to existing approaches.

**Table 4.** Comparison of average object detection accuracy between the proposed method and other methods in the KITTI dataset.

| Method | Input | AP3D _R11(%) | | APBEV _R11(%) | | | | |
| --- | --- | --- | --- | --- | --- | --- | --- | --- |
|  |  | Easy | Moderate | | Hard | Easy | Moderate | Hard |
| VoxelNet | LiDAR | 67.17 | 47.65 | | 45.11 | 74.41 | 52.18 | 50.49 |
| PillarNet | LiDAR | 77.41 | 58.09 | | 55.69 | 80.85 | 62.63 | 59.19 |
| SECOND | LiDAR | 76.34 | 62.20 | | 58.50 | 81.62 | 68.58 | 63.65 |
| PointPillars | LiDAR | 78.71 | 60.83 | | 57.45 | 83.04 | 65.51 | 62.48 |
| MAT-PointPillars | LiDAR | 81.15 | 62.02 | | 58.68 | 83.85 | 66.38 | 62.63 |

As Table 4 indicates, the proposed MAT-PointPillars algorithm achieves an AP3D_R11(%) of 81.15%, 62.02%, and 58.68% on easy, moderate, and hard samples, which are higher than the benchmark model by 2.44%, 1.19%, and 1.23% respectively. The algorithm obtains 83.85%, 66.38%, and 62.63% of easy, moderate, and hard samples in the BEV, which are higher than the benchmark model 0.81%, 0.87%, and 0.15%, respectively. Compared with VoxelNet, PillarNet, the AP3D_R11(%) and APBEV_R11(%) of MAT-PointPillars are higher than those of VoxelNet and PillarNet in easy, moderate, and hard tasks. Compared with SECOND, AP3D_R11(%) in easy and hard samples are higher than SECOND by 4.81%, 0.18% respectively. Our algorithm mainly focuses on 3D object detection, so AP3D_R11(%) has good indicators. The algorithm achieves superior detection performance.

The updated results and discussions are now included in the revised manuscript. We believe these additions significantly strengthen the paper and offer readers a clearer understanding of the advancements achieved by our proposed method.

2. **Is the chart in the article in the wrong place? Why are they all placed at the end of the article? /Unreasonable placement of figures.**

**Reply:** Thank you very much for your suggestion. The position of the chart in the text is important to the reader's reading experience and understanding. Here is a detailed explanation of where the chart is: In PLOS ONE's submission guidelines, the submission requirement for figures is “Do not include figures in the main manuscript file. Each figure must be prepared and submitted as an individual file.” So, in this article, the figures will be placed centrally at the end of the article.

We understand that reviewers may prefer to embed the chart in the text so that readers can more easily view the figure as they read. We fully agree on the advantages of this approach, especially when the number of figures is small, or the figure is closely related to the content of the text. If the reviewer thinks it is more appropriate to embed the figure in the article, we can adjust the position of the figure in the revision, inserting each figure near the position first mentioned in the article. This will further improve the readability and coherence of the article.

# Reviewer #1:

1. **Figure 9 is a screenshot.**

**Reply:** Thank you very much for your suggestion. We sincerely apologize for the oversight in including Figure 9 as a screenshot. This was unintentional and does not meet the standards expected for publication. We have replaced the screenshot with a high-quality, version of the figure in the paper.

2. **The article lacks an analysis of ablation experiments regarding why CBAM achieves higher detection accuracy compared to CA.**

**Reply:** Thank you very much for your suggestion. To explain why CBAM (Convolutional Block Attention Module) is superior to CA (Coordinate Attention) in detection accuracy, it can be analyzed from the following aspects and discussed in detail combined with the results of ablation experiments:

First, CBAM can enhance feature representation in both channel and space through the attention mechanism of these two dimensions. The channel dimension is unchanged, and the space dimension is compressed. This module focuses on meaningful information in the input image.CA focuses on coordinate information and captures spatial position information by decomposing global pooling into pooling operations in the direction of width and height. This module is concerned with the location information of the target. The advantage of CA is that it can better capture the location information of the target, but the attention mechanism in the channel dimension is weak. Furthermore, in 3D object detection tasks, the size and shape of objects vary greatly, and CBAM can better capture these changes through channels and spatial attention mechanisms, thereby improving detection accuracy.

According to the results of ablation experiments, CBAM is superior to CA in detection accuracy, as shown in Table 5. Specifically, CBAM outperformed CA on both AP3D and APBEV. For example, the combination of CBAM+CBAM reached 81.15% on AP3D, while the combination of CA+CA was only 73.47%.

**Table 5.** Results of ablation experiment

| Attention | Batch | Head | Layer | AP3D | APBEV |
| --- | --- | --- | --- | --- | --- |
| CBAM+CBAM | 2 | 2 | 1 | 78.40% | 81.07% |
| CBAM+CBAM | 2 | 4 | 1 | 76.41% | 79.93% |
| CA+CA | 2 | 4 | 2 | 73.47% | 78.91% |
| CA+CBAM | 2 | 4 | 2 | 74.98% | 78.00% |
| CBAM+CBAM | 2 | 4 | 2 | 81.15% | 83.85% |

CBAM's attention mechanism makes it superior to CA in multi-scale feature extraction, complex scene robustness and small target detection. Experimental data verified its effectiveness. The relevant content has been revised and supplemented in the paper.

# Reviewer #2:

1. **Inconsistent tenses throughout the paper.**

**Reply:** Thank you very much for your suggestion. For the problem of inconsistent tenses, we have made the following adjustments in the revised draft:

Algorithm description: unified use of the present tense. For example, change "The algorithm improved the accuracy" to "The algorithm improves the accuracy".

Experiment part: Unified use of present tense. For example, replace "The model achieved an accuracy of 81.15%" with "The model achieves an accuracy of 81.15%".

Conclusion and future work section: Use present or future tense. For example, replace "We proposed future work" with "We propose future work" or "We will propose future work".

In addition to tense issues, we also thoroughly polish the language of the article to ensure that the expression is clear, accurate, and meets the norms of academic writing. We pay special attention to the problems of verb tense, subject-verb agreement, sentence structure, etc., to ensure that the language quality. The relevant content has been revised in the paper.

2. **Experiments are conducted on only one dataset, which lacks credibility.**

**Reply:** Thank you very much for your suggestion. We appreciate the reviewer's valuable feedback regarding the need for experiments on multiple datasets to enhance the credibility of our results. The KITTI dataset is widely recognized as a standard benchmark for 3D object detection in autonomous driving scenarios. It provides a comprehensive evaluation framework with multiple difficulty levels (easy, moderate, and hard), which allows for a thorough assessment of the algorithm's performance under various conditions. Many state-of-the-art methods in 3D object detection, including VoxelNet, PillarNet, SECOND and PointPillars, have been extensively evaluated on the KITTI dataset. By comparing our results with these methods, we ensure that our algorithm's performance is competitive and well-validated within the context of this widely accepted benchmark.

The primary focus of this study was to introduce and validate the effectiveness of the multi-scale attention mechanisms and Transformer Encoder in improving the PointPillars algorithm. The KITTI dataset provided a suitable platform for this purpose, as it allowed us to conduct detailed ablation studies and compare our results with existing methods.

In conclusion, while we recognize the importance of evaluating our algorithm on multiple datasets, we believe that the current results on the KITTI dataset provide meaningful insights into the effectiveness of our proposed method. We are committed to extending our experiments to other datasets in future work to further validate the generalization ability of our approach.

3. **Lack of exploration of the effect of the number of layers on performance in the ablation experiments.**

**Reply:** Thank you very much for your suggestion. In our experiments, the number of Transformer Encoder layers was set to 2, which is the maximum supported by our current experimental environment (NVIDIA RTX 3060 Ti GPU). Increasing the number of layers would lead to a significant increase in GPU memory usage, exceeding the GPU's memory capacity. As the number of Transformer Encoder layers increases, the computational complexity of the model also increases significantly, leading to longer inference times and negatively impacting real-time performance.

In our experiments, the 2-layer Transformer Encoder already provides high detection accuracy while maintaining real-time performance (average frame rate of 22.63 fps). Increasing the number of layers might further improve accuracy but would significantly degrade real-time performance. Experiments show that the balance between accuracy and efficiency of 2-layer Transformer Encoder is the optimal solution under current hardware conditions. The effect of the number of layers on algorithm performance has been supplemented and added in the paper.
